# Impact of energy drink versus coffee consumption on periodic repolarization dynamics: an interventional study

**DOI:** 10.1007/s00394-022-02853-8

**Published:** 2022-03-10

**Authors:** Dominik Schüttler, Wolf-Stephan Rudi, Axel Bauer, Wolfgang Hamm, Stefan Brunner

**Affiliations:** 1 Department of Medicine I, University Hospital, LMU Munich, Marchioninistrasse 15, 81377 Munich, Germany; 2 German Center for Cardiovascular Research (DZHK), Partner Site Munich, Munich Heart Alliance (MHA), 80802 Munich, Germany; 3 Institute of Surgical Research at the Walter-Brendel-Centre of Experimental Medicine, University Hospital, LMU Munich, Marchioninistrasse 27, 81377 Munich, Germany; 4 University Hospital for Internal Medicine III, Medical University Innsbruck, Innsbruck, Austria

**Keywords:** Autonomic dysfunction, Periodic repolarization instability, Caffeine, Energy drink, Coffee

## Abstract

**Purpose:**

Caffeinated beverages are consumed daily throughout the world. Caffeine consumption has been linked to dysfunction of the autonomic nervous system. However, the exact effects are still insufficiently understood.

**Methods:**

Sixteen healthy individuals were included in the present non-randomized cross-over interventional study. All study subjects consumed a commercial energy drink (containing 240 mg caffeine), and in a second independent session coffee (containing 240 mg caffeine). High-resolution digital ECGs in Frank-lead configuration were recorded at baseline before consumption, and 45 min after consumption of the respective beverage. Using customized software, we assessed ECG-based biomarker *periodic repolarization dynamics* (PRD), which mirrors the effect of efferent cardiac sympathetic activity on the ventricular myocardium.

**Results:**

The consumption of energy drinks resulted in an increase in PRD levels (3.64 vs. 5.85 deg^2^; *p* < 0.001). In contrast, coffee consumption did not alter PRD levels (3.47 vs 3.16 deg^2^, *p* = 0.63). The heart rates remained unchanged both after coffee and after energy drink consumption. Spearman analysis showed no significant correlation between PRD changes and heart rate changes (*R* = 0.34, *p* = 0.31 for coffee, *R* = 0.31, *p* = 0.24 for energy drink).

**Conclusion:**

Our data suggests that sympathetic activation after consumption of caffeinated beverages is independent from caffeine and might be mediated by other substances.

**Trial Number:** NCT04886869, 13 May 2021, retrospectively registered

## Introduction

Consumption of caffeinated drinks has a long cultural and social tradition throughout the world making caffeine the most widely used psychoactive and stimulating agent [1]. Both, deleterious and beneficial health effects have been attributed to caffeine consumption: in the past, major concerns have been raised about potential systemic adverse effects of a long-term consumption of caffeinated beverages including the development of cancer, arrhythmias and other cardiovascular diseases [2, 3]. In contrast, a large prospective study showed that coffee consumption—one of the most widely consumed caffeine containing beverages—was inversely associated with total and cause-specific mortality [4]. However, these contrary research findings should be interpreted with caution, since caffeinated beverages contain many other biologically active substances, and therefore, health effects may not only be related to caffeine [3, 5].

From the cardiovascular point of view, caffeinated beverages may contribute to the development of arrhythmias [5, 6]. The underlying mechanisms of arrhythmogenesis are not completely understood. One potential mechanism is the caffeine-induced imbalance of the autonomic nervous system (ANS). Various studies intended to support this hypothesis by demonstrating changes of heart rate variability, a measure of the balance of the ANS [7]. However, results remain still inconclusive.

Periodic repolarization dynamics (PRD) is a novel ECG-based biomarker, which most likely reflects the activity of the sympathetic branch of the ANS on the level of the ventricular myocardium [8]. It has proven its strong prognostic power in large trials of patients with ischemic and non-ischemic cardiomyopathy [8, 9]. Several studies demonstrated its regulation among physiological states which are known to activate the sympathetic nervous system [10–12]. However, the effect of caffeine consumption on PRD levels and on cardiac repolarization instability, is still insufficiently explored. Thus, the aim of the present pilot study was to investigate the effect of consumption of caffeinated beverages on PRD.

## Methods

### Participants

For the present study, 16 healthy individuals (6 females and 10 males) with a mean age of 30.2 ± 7.9 years (standard deviation) were recruited between March and May 2021. Subjects with any previous history of cardiovascular disease or on daily medication were excluded from the study. Table 1 provides further information about baseline characteristics.

**Table 1:** Overview of baseline characteristics. Values show mean ± standard deviation

	Total (*n* = 16)	Male (*n* = 10)	Female (*n* = 6)
Age (years)	30.2 ± 7.9	30.7 ± 5.5	29.3 ± 10.8
Height (cm)	176.4 ± 8.3	181.8 ± 5.0	167.3 ± 3.5
Weight (kg)	75.1 ± 11.8	78.9 ± 11.1	68.7 ± 10.2
BMI (kg/m^2^)	24.1 ± 3.4	23.8 ± 2.7	24.6 ± 4.2

### Ethics

The study protocol was approved by the local ethics committee (*“Ethikkommission der Medizinischen Fakultät der LMU München”,* project no. 370–16). Informed consent was obtained from each patient, and the study protocol conforms to the ethical guidelines of the Declaration of Helsinki.

### Sample size

As there is no data available regarding the effect of caffeine on PRD levels, we estimated sample size by looking at comparable studies which investigated the effect of caffeine on HRV [13, 14]. PRD has been proven to be a highly sensitive parameter to monitor sympathetic nervous system on the level of ventricular myocardium [8, 15]. Previous studies of our group with similar sample sizes showed robust statistical differences on PRD changes in response to triggers stimulating the sympathetic nervous system [10–12]. We thus assumed that our number of participants would be sufficient to see possible effects.

### Design

In this cross-over intervention study, each participant took part in two sessions in a randomized order. There was an interval of at least 48 h between the sessions. In session 1, study participants consumed 750 mL of a commercial energy drink (containing 32 mg caffeine/100 mL (0.03%), 0.4% taurine, 11 g/100 ml sugar according to the manufacturer’s information). In session 2, study participants consumed three cups of coffee (containing 80 mg caffeine/cup according to the manufacturer’s information). Volunteers were urged to consume drinks within 15 min time. In each session, a baseline high-resolution (1000 Hz) digital 20-min ECG (Schiller medilog AR4plus; Schiller AG, Switzerland) was performed in Frank-lead configuration in a resting sitting position, not talking, and quiet surroundings. After baseline recordings, the respective beverage was consumed within 5 min. Caffeine absorption is known to be completed approximately 45 min after intake and blood levels peak around that time [16]. Therefore, after another resting period of 45 min, a second 20-min high-resolution ECG was recorded. Study participants were required to abstain from caffeine and alcohol consumption 48 h before each session. To exclude volume effects of drinks on PRD changes, we performed an additional experimental session, where study participants drank 750 ml of tap water, and calculated PRD before and after water intake.

All data were collected and analyzed by the study investigators at the Department of Cardiology at the University Hospital Munich (LMU). PRD was calculated out of ECG recordings by a blinded investigator.

### Outcome measures

PRD was calculated as described [8]. In brief, the spatiotemporal properties of each T wave were integrated into a single vector (T°). The angle between two successive vectors (dT°) is plotted over time representing the instantaneous degree of repolarization fluctuations. Typically, an oscillating pattern of the dT° signals can be observed. PRD is calculated by use of wavelet analyses in the low-frequency spectrum (≤ 0.1 Hz).

ECG signals were analyzed using the MATLAB software with established algorithms for calculation of PRD.

### Statistics

Results are expressed as median and corresponding interquartile range (IQR). Statistical analyses were performed with R version 4.0.3. Wilcoxon signed rank test was used to detect differences in PRD levels and heart rates in response to coffee as well as ED consumption (*p* < 0.05 estimated as statistical significance). Spearman correlation analysis were performed to test the correlation between changes in heart rate and changes in PRD levels.

## Results

**Fig. 1:**
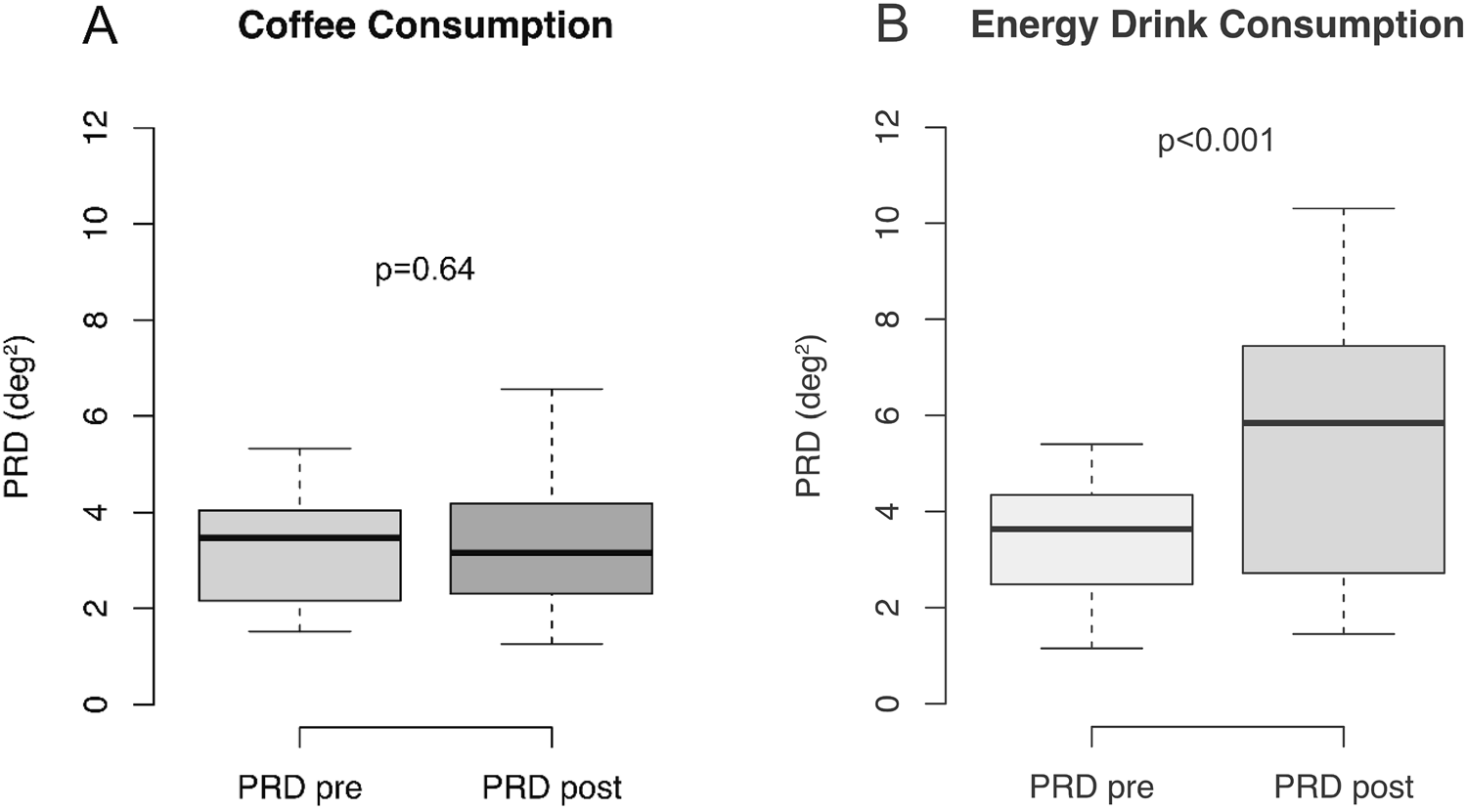
Boxplots show changes in PRD levels after coffee consumption **(A)** and energy drink consumption **(B)**. Boxes visualize medians with interquartile ranges. Wilcoxon signed rank tests were performed to detect statistical differences (*p* < 0.05)

After coffee consumption PRD values did not change significantly compared to baseline values (*p* = 0.64): PRD at baseline was 3.47 deg^2^ (IQR 1.88 deg^2^) and remained unchanged at 3.16 deg^2^ (IQR 1.89 deg^2^) after coffee consumption (Fig. 1A). Additionally, mean heart rate did not change when comparing values before and after coffee intake (80.8 bpm (IQR 5.4 bpm) vs. 75.0 bpm (IQR 5.7 bpm); *p* = 0.15). There was no significant correlation in Spearman analysis between changes in heart rates and changes in PRD levels (Spearman’s rank correlation coefficient *R* = 0.34, *p* = 0.31).

In contrast to coffee consumption, PRD values increased significantly after consumption of energy drinks from 3.64 deg^2^ (IQR 1.80 deg^2^) at baseline to 5.85 deg^2^ (IQR 4.40 deg^2^) (*p* < 0.001; Fig. 1B). Mean heart rate did not change significantly after ED consumption (78.9 bpm (IQR 7.8 bpm) before vs. 80.4 bpm (IQR 9.8 bpm) after intake; *p* = 0.56) and we did not detect a significant correlation between PRD changes and changes in heart rate (Spearman’s rank correlation coefficient *R* = 0.31, *p* = 0.24). Consumption of tap water (750 ml) did not alter PRD levels (2.94 deg^2^ (IQR 0.85 deg^2^) vs. 2.77 deg^2^ (IQR 1.1 deg^2^), *p* = 0.79).

## Discussion

In our study, we investigated the acute effect of consumption of caffeinated beverages on the sympathetic activity by means of analysis of PRD levels. For this purpose, we have chosen two popular caffeine containing beverages, coffee and energy drinks. We detected a significant increase of PRD levels after consumption of energy drinks indicating enhanced efferent cardiac sympathetic activity. In contrast, we did not observe an effect on PRD levels after coffee consumption. Water consumption did not change PRD levels, excluding an effect on PRD in response to volume intake.

A previous study investigated the effect of energy drink consumption on standard electrocardiographic and blood pressure parameters. The authors described a significant increase of systolic blood pressure after energy drink consumption indicating an activation of the sympathetic branch of the autonomic nervous system [7]. In another study by this group, the authors compared the effect of energy drinks with another caffeinated beverage. The systolic blood pressure was significantly higher with energy drinks [17]. A study by Corti et al. detected an increased muscle sympathetic nerve activity after coffee consumption. However, in this study, the sympathetic activity was likewise activated in caffeinated and decaffeinated coffee. Thus, the observed effect was independent from caffeine [18].

In line with these studies, our findings on changes in PRD levels suggest, that the effect of caffeinated beverages on sympathetic activity-mediated cardiac repolarization instability is independent from caffeine and is mediated by other substances. The ingredients of caffeinated beverages are various. It is well known that energy drinks contain additional energy-boosting substances, such as taurine, guarana, and sugar. These substances may affect the cardiovascular system and may lead to a proarrhythmic substrate via sympathetic activation [3, 5]. There are several reports linking an overuse of energy drinks to the occurrence of sudden cardiac deaths [5, 6]. This risk may be reflected by the elevated PRD levels after consumption of energy drinks, since PRD is an excellent predictor of mortality and in particular of sudden cardiac deaths in patients with underlying heart diseases [8, 9].

A number of considerations are necessary when interpreting our results. With a single ECG recording, we cannot elucidate the temporal relation between caffeine consumption and PRD changes. Further, we did not investigate dose dependent effects of caffeine on PRD levels. In addition, we cannot exclude individual differences of caffeine absorption, since we did not measure blood caffeine levels. Results are limited by the small sample size, further studies investigating the effect of caffeine on PRD, e.g. in combination with HRV-derived parameters might be useful to further support our findings.

In conclusion, our results suggest that sympathetic activity-mediated repolarization instability after consumption of caffeinated beverages is independent from caffeine and might be triggered by other substances.

## Authors` contributions

DS collected, analyzed and interpreted data and wrote the original draft. WSR collected data and revised the manuscript for intellectual content. AB revised the manuscript for intellectual content. WH collected, analyzed and interpreted data and revised the manuscript for intellectual content SB was responsible for conceptualization and project administration and revised the manuscript for intellectual content. All author’s read the manuscript and agreed to its submission.

## Declarations

### Availability of data and materials

All data sets can be obtained from the corresponding author by request.
